# Epstein–Barr Virus (EBV) Genotypes Associated with the Immunopathological Profile of People Living with HIV-1: Immunological Aspects of Primary EBV Infection

**DOI:** 10.3390/v14020168

**Published:** 2022-01-18

**Authors:** Leonn Mendes Soares Pereira, Eliane dos Santos França, Iran Barros Costa, Igor Tenório Lima, Amaury Bentes Cunha Freire, Francisco Lúzio de Paula Ramos, Talita Antonia Furtado Monteiro, Olinda Macedo, Rita Catarina Medeiros Sousa, Felipe Bonfim Freitas, Igor Brasil Costa, Antonio Carlos Rosário Vallinoto

**Affiliations:** 1 Laboratory of Virology, Institute of Biological Sciences, Federal University of Pará (UFPA), Belém 66075-110, PA, Brazil; leonnpereira@hotmail.com; 2 Epstein-Barr Virus Laboratory, Virology Department, Evandro Chagas Institute (IEC), Ananindeua 67030-000, PA, Brazil; elianesantos_85@hotmail.com (E.d.S.F.); irancosta@iec.gov.br (I.B.C.); igortenorio003@gmail.com (I.T.L.); talitamonteiro@iec.gov.br (T.A.F.M.); igorcosta@iec.gov.br (I.B.C.); 3 Epidemiology and Surveillance Service, Evandro Chagas Institute (IEC), Ananindeua 67030-000, PA, Brazil; amaurybentes@iec.gov.br (A.B.C.F.); franciscoramos@iec.gov.br (F.L.d.P.R.); 4 Retrovirus Laboratory, Virology Department, Evandro Chagas Institute (IEC), Ananindeua 67030-000, PA, Brazil; olindamacedo@iec.gov.br (O.M.); felipefreitas@iec.gov.br (F.B.F.); 5 School of Medicine, Federal University of Pará (UFPA), Belém 66075-110, PA, Brazil; ritaclosset@uol.com.br; 6 Graduate Program in Virology, Evandro Chagas Institute (IEC), Ananindeua 67030-000, PA, Brazil; 7 Graduate Program in Biology of Infectious and Parasitic Agents, Institute of Biological Sciences, Federal University of Pará (UFPA), Belém 66075-110, PA, Brazil

**Keywords:** HIV-1, EBV, coinfection, immunopathological profile

## Abstract

Background: The aim of the present study was to evaluate the immunological profile of adult HIV-1^+^ patients coinfected with primary Epstein–Barr virus (EBV) infection who were free of antiretroviral drugs and inhabitants of the Brazilian Amazon region. Materials and methods: Primary EBV infection was screened by the semiquantitative detection of IgM and IgG anti-VCA. Genotypes were determined by conventional PCR. EBV and HIV viral load (VL) were quantified by real-time PCR. Cytokine dosage and cell quantification were performed by cytometry. Results: Only HIV-1^+^ individuals had primary EBV infection (7.12%). The EBV-1 genotype was the most prevalent (47.37%). The VL of HIV-1 was lower in the HIV/EBV-2 group. CD4^+^ T lymphocytes were inversely proportional to the VL of EBV in HIV/EBV-1/2 multi-infected patients. The HIV/EBV-2 group had the lowest cytokine levels, especially IFN-γ and IL-4. Different correlations were proposed for each coinfection. The late search for specific care related to HIV infection directly affected the cytokine profile and the number of CD8^+^ T lymphocytes. Symptoms were associated with the increase in VL of both viruses and cytokine profile. Conclusions: Different immunological profiles were associated with EBV genotypes in primary infection, with EBV-2 being more frequent in patients with low levels of HIV viral load. With late infection monitoring and consequent delay in the initiation of HAART, clinical changes and effects on the maintenance of the immune response were observed.

## 1. Introduction

Epstein–Barr virus (EBV) is considered one of the risk factors for morbidity in people living with HIV (PLHIV) because it is a precursor of neoplasms in this group [1,2,3]. Coinfection is related to progressive dysfunction events and impaired immune surveillance, which becomes critical when progressing to AIDS, in which severe immunosuppression results in pathological signs of malignant neoplasms [4,5,6].

In primary EBV infection, the pathological manifestations of coinfection are more studied in children, in whom HIV and persistent EBV replication results in a severe spectrum of clinical symptoms, including splenomegaly, hepatomegaly, and pneumonia, which increase the risk of hospitalization recurrence [7,8].

The discussion becomes more complex when considering that the prevalence of EBV genotypes may be associated with the immune profile of the host coinfected with HIV. EBV-1 is more prevalent in patients with fewer helper T lymphocytes and a high HIV viral load, while EBV-2 predominates in more responsive immune profiles comprising numerous helper T lymphocytes and a low HIV viral load [9,10,11]; However, it is not clear whether this trend is a pattern observed only in primary EBV infection.

In this context, the aim of this study was to evaluate the immunological profile of adult PLHIV who were free of antiretroviral drugs (HAART) and presented with primary coinfection with different EBV genotypes; we sought to associate immunological markers with the viral dynamics of HIV/primary EBV coinfection and the clinical aspects of the patients to reveal the possible impacts of the late attendance of the patient.

In developing countries, primary EBV infection is more frequent in childhood, especially in children of low socioeconomic status [12,13], which presents a challenge regarding the aims of this study. However, we emphasize that there are few studies on HIV/primary EBV coinfection in the Brazilian Amazon and the northern region of the country; therefore, our findings may contribute to discussions on the epidemiological scenario of HIV/primary EBV coinfection in Brazil, as well as on the complex viral interaction intrinsic to the context of coinfection.

## 2. Materials and Methods

### 2.1. Sampling

This was an observational, cross-sectional, and analytical study conducted at the Laboratory of Virology at the Federal University of Pará (LABVIR-UFPA), the Evandro Chagas Institute (IEC), the Center for Health Care in Acquired Infectious Diseases (CASA DIA, for its acronym in Portuguese), and the Hemotherapy and Hematology Foundation of the State of Pará (HEMOPA, for its acronym in Portuguese), where 20 weekly samples from participants living in the state of Pará, Brazil, were selected from January 2018 to December 2019.

Samples were collected from 333 participants who were categorized as 249 PLHIV no primary EBV infection (p24+, anti-HIV-1 IgG [+], anti-VCA-EBV IgM [−], and anti-VCA-EBV IgG [+ or −]), 19 primary EBV and HIV-1 coinfected (p24+, anti-HIV-1 IgG [+], anti-VCA-EBV IgM [+], and anti-VCA-EBV IgG [−]); confirmation by molecular biology assay), and 65 no HIV-1 infection or primary EBV infection (p24[−], anti-HIV-1 IgG [−], anti-VCA-EBV IgM [−], and anti-VCA-EBV IgG [+ or −]). All participants answered an epidemiological questionnaire administered at the time of the interview.

The project was submitted for ethical review and approved by the Human Research Ethics Committee of the IEC (Protocol: 3.121.265; CAAE: 73927717.3.0000.0019). All participants were informed about the study objectives and signed an informed consent form.

Participants included in the study were 18 years old or older, of both sexes, and HIV-1 and/or EBV carriers, were not using antiviral therapy, and signed the informed consent form.

### 2.2. Confirmatory Methods

Peripheral blood samples were collected from the participants. DNA was extracted from the plasma and buffy coat using a QiaAmp DNA Mini Kit (Qiagen, Düsseldorf, Nordrhein-Westfalen, Germany) following the manufacturer’s recommendations.

Samples were screened for HIV-1 infection by the qualitative detection of p24 antigen and anti-HIV-1 and anti-HIV-2 IgG antibodies using an enzymatic immunoassay (Murex AG/AB Combination DiaSorin, Dartford, Kent, UK); serological confirmation was performed using the Immunoblot rapid DPP HIV-1/2 kit (Bio-Manguinhos, Rio de Janeiro, RJ, Brazil) following the manufacturer’s recommendations.

Infection by EBV was screened by the semiquantitative detection of anti-EBV IgM and IgG antibodies using an enzymatic immunoassay (Ridascreen EBV VCA R-Biopharm, Darmstadt, Hesse, Germany). The identification of the EBV genotypes was performed by nested PCR targeting the EBNA-3C gene using the primers described by Lorenzetti et al. [14] (1st round–(F: 5′-AGATGGTGAGCCTGACGTG-3′/R: 5′-GCATCCTTCAAAACCTCAGC-3′) and Sample et al. [15] (2nd round–F: 5′-AGAAGGGGAGCGGTGTGTTGT-3′/R: 5′-GGCTGTTTTTGACGTCGGC-3′). The reaction components and cycling conditions were as follows: 10 pmol/µL of primers, MgCl_2_ (50 mM), dNTP (10 mM), and Taq (5 U/µL); 1st round–1 cycle of 95 °C/3′, 20 cycles of (94 °C/45″; 56 °C/45″; and 72 °C/45″), and 1 cycle of 72 °C/7′; and 2nd round–1 cycle of 95 °C/3′; 35 cycles of (94 °C/45″; 56 °C/45″; and 72 °C/45″); and 1 cycle of 72 °C/7′. The presence of a 153 bp fragment was considered positive for EBV-1, and the presence of a 246 bp fragment was considered positive for EBV-2.

### 2.3. Quantification of T Lymphocytes and Measurement of Cytokines

The quantification of T helper (CD45^high^CD3^+^CD4^+^CD8^−^), T cytotoxic (CD45^high^CD3^+^CD4^−^CD8^+^), double positive (DP) (CD45^high^CD3^+^CD4^+^CD8^+^), and double negative (DN) (CD45^high^CD3^+^CD4^−^CD8^−^) lymphocytes was performed by immunophenotyping and flow cytometry using BD FACSCalibur (4 colors) equipment, FACSCount^TM^ Reagents, and TriTEST™/TruCount monitoring kits (BD Biosciences, San Jose, CA, USA). We used the BD Multiset™ Software v3.1 software (BD Biosciences, San Jose, CA, USA) already standardized for analysis of lymphocyte populations related to HIV infection.

The plasma concentrations of the cytokines IL-17A, IFN-γ, TNF, IL-10, IL-6, IL-4, and IL-2 were determined with a cytometric bead array (CBA) using BD FACSCanto™ II equipment and a BD^TM^ CBA Human Th1/Th2/Th17 Cytokine kit (BD Biosciences, San Jose, CA, USA).

### 2.4. Quantification of HIV-1 and EBV Viral Loads

The quantification of the HIV-1 plasma viral load was performed by real-time PCR using an Abbott Sample Preparation System RNA extraction kit and the Abbott Real-Time HIV-1 amplification matrix (ABBOTT, Chicago, IL, USA).

The quantification of the EBV viral load was based on an estimation matrix by real-time PCR, following the protocol of the XGEN MASTER EBV kit (Mobius Life Science, Pinhais, PR, Brazil). We used buffy coat and plasma samples to quantify the viral load.

### 2.5. Statistical Analysis

In the sample characterization, we compared sociodemographic data, spontaneous delay in seeking specific care (late attendance) and symptomatology among groups using the G test. Fisher’s exact test was used exclusively for data dispersed in 2 × 2 tables.

We applied the Kruskal–Wallis test for the analysis of variance in viral loads, number of lymphocytes and cytokine levels among the groups studied; we opted for the Student-Newman–Keuls method as a complement to compare the ranked means.

We adopted the same methodology in the assessment of immunological changes based on the time of delay in seeking specific care and symptomatology. We chose a nonparametric test after determining that the distribution of the samples did not meet the assumption of normality, as calculated by the Lilliefors test.

The Spearman coefficient was calculated in the correlation of the EBV viral loads quantified in different blood extracts with the other factors studied. We used curve fitting to determine the correlation between viral loads and the correlation between the EBV viral load and the number of lymphocytes.

Correlation matrices between all immunological and virological factors analyzed were proposed for the group of PLHIVs without primary EBV infection and for the coinfected groups based on Pearson’s coefficient (r).

A dispersion diagram was inferred based on multivariate discriminant analysis of the immunological factors and viral load of the studied groups.

The statistical analyses were performed using GraphPad Prism 3.03 (San Diego, CA, USA) and BioEstat 5.0 [16].

## 3. Results

### 3.1. Sample Characterization

Of the 333 participants evaluated between January 2018 and December 2019, only 19 were positive for primary EBV infection, all of them infected with HIV-1; we obtained a prevalence of 7.09% of coinfected individuals (Table 1), with the majority living in the metropolitan mesoregion of Belém city (98.20%). 

All 65 uninfected individuals were healthy blood donors with no history of comorbidities. The 249 PLHIV no primary EBV infection and the 19 HIV/primary EBV coinfected were outpatients with a positive pre-diagnosis for HIV with or without a history of associated comorbidities (Table 1). Both uninfected and PLHIV no primary EBV infection had positive anti-VCA IgG serology, indicating previous contact with the EBV.

Among those coinfected, the EBV-1 genotype was the most prevalent (47.37%), followed by the EBV-2 genotype and multi-infection with both genotypes, both of which occurred in the same proportion in the population (26.32%) (Table 1). There was a predominance of male individuals aged 18 to 38 years in all groups, except for HIV/primary EBV-1 coinfected patients, among whom women were more prevalent (Table 1).

### 3.2. Evaluation of the EBV Viral Load in Different Blood Extracts and Its Correlation with HIV-1 Viral Load

**Figure 1:**
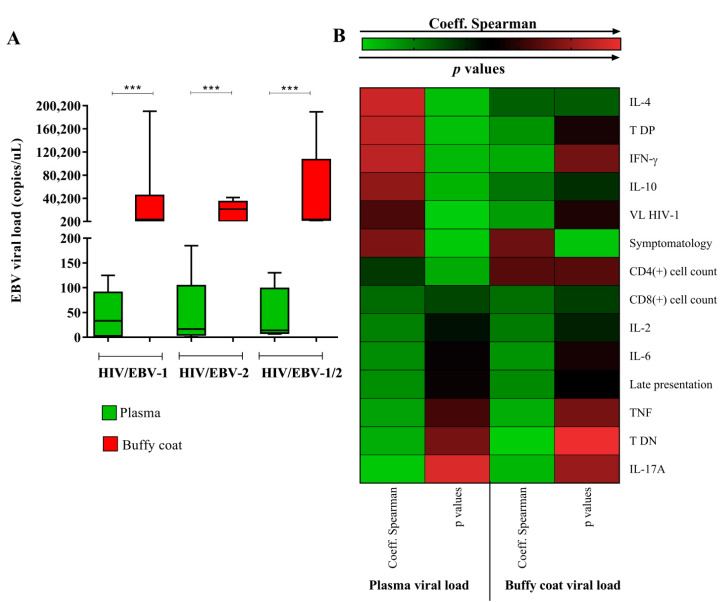
Analysis of the EBV viral load in different blood extracts. **(A)** Graph showing the quantification of the EBV viral load in the plasma and buffy coat of the HIV/primary EBV coinfected groups. ***: *p* < 0.0001. (**B**) Heatmap illustrating the absolute values of the Spearman coefficient and respective *p* value for the correlations between the EBV viral load quantified in plasma and in the buffy coat with the other factors studied. The most significant correlations were observed for plasma viral load; in the buffy coat, the viral load was associated only with the patients’ symptoms. The statistical data for each correlation are detailed in Supplementary Table S1.

We evaluated which blood extract would be the most appropriate for the quantification of EBV viral load in primary infection. We observed that the biomarker in the buffy coat was considerably higher than in the blood plasma [*p* < 0.0001; (HIV/EBV-1 plasma: median: 33.33 IQ (25–75%): 6–67.33); (HIV/EBV-1 buffy coat: median: 3668 IQ (25–75%): 1663.33–4793.33); (HIV/EBV-2 plasma: median: 16.67 IQ (25–75%): 8.67–23.33); (HIV/EBV-2 buffy coat: median: 21,322.67 IQ (25–75%): 970.67–27,473.33); (HIV/EBV-1/2 plasma: median: 14 IQ (25–75%): 10–66.67); (HIV/EBV-1/2 buffy coat: median: 3591.33 IQ (25–75%): 3562–24,485.33)] (Figure 1A). However, when calculating the correlation of the viral load with the other markers studied, we observed that the plasma viral load generated the most significant correlations (Figure 1B); therefore, plasma was chosen as the blood extract for the subsequent analyses. In Table S1, we detail the statistical data referring to the Spearman coefficient (rs) and respective probability value (*p*) for each proposed correlation. Notably, only the symptoms of the patients were associated with the quantified viral load in both blood extracts (rs plasma: 0.55; *p*: 0.015/rs buffy coat: 0.52; *p*: 0.022). A priori, this first analysis was focused only on the choice of the ideal sample for the quantification of the viral load; the biological significance of the associations generated was explored throughout the study.

In an intergroup analysis, the median plasma viral load in the HIV/EBV-1 group was higher (median: 33.33 (IC 95%): 6–67.33) but not statistically significant (*p*: 0.962) (Figure 1 A). 

**Figure 2:**
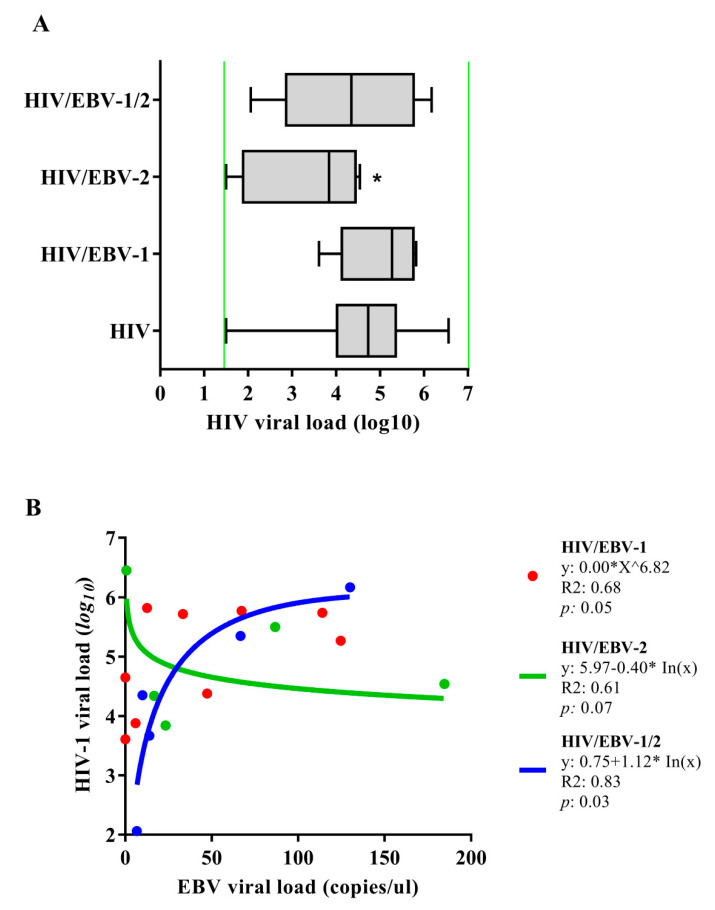
Quantification of viral load: (**A**) Quantification of the HIV-1 plasma viral load. Green lines indicate the limits of detection for the assay used (<log1.6 (40 copies)—>log7 (10,000,000 copies)); *: *p* < 0.05. (**B**) Regression graph for viral loads of HIV-1 and EBV.

The median log10 of the HIV-1 viral load was lower in the HIV-EBV-2 group when compared to the others [*p*: 0.0207; (HIV: med: 4.73 IQ (25–75%): 4.03–5.35); (HIV/EBV-1: med: 5.27 IQ (25–75%): 4.38–5.74); (HIV/EBV-2: med: 3.84 IQ (25–75%): 2.27–4.34); (HIV/EBV-1/2: med: 4.35 IQ (25–75%): 3.67–5.34)] (Figure 2A).

We conducted a regression analysis of the viral loads of both viruses in the coinfected patients (Figure 2B). In the HIV/EBV-1 group, the geometric regression indicated a trend between the markers (*p*: 0.05; R2: 0.68), in which the EBV-1 viral load was distributed in a symmetrical curve with a peak of 125 copies/µL, followed by a decrease as the log10 of the HIV-1 viral load increased. We found an inversely proportional relationship between the viral loads in the HIV/EBV-2 group that was best represented by a decreasing logarithmic regression (*p*: 0.07; R2: 0.61) in which the HIV-1 viral load slowly decreased as the EBV-2 viral load increased. In contrast, we observed the opposite for the HIV/EBV-1/2 multi-infected group; the regression between viral loads was directly proportional and represented by an increasing logarithmic function (*p*: 0.03; R2: 0.83). In this case, the HIV-1 viral load gradually increased as the viral loads of EBV-1 and EBV-2 increased.

### 3.3. Different Immunological Profiles Were Associated with the EBV Genotypes

**Figure 3:**
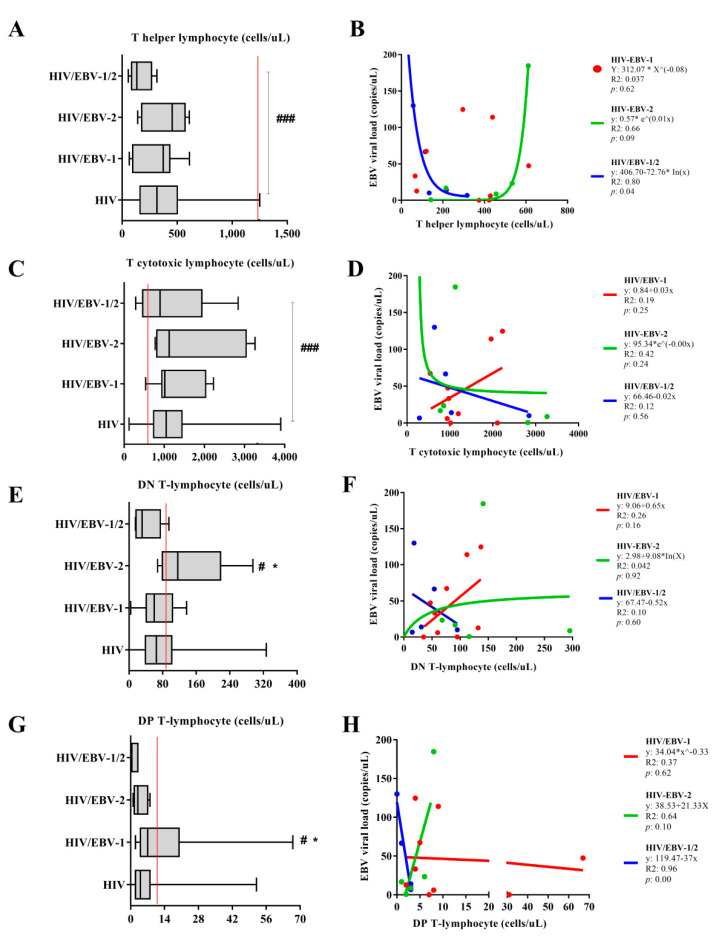
Cell quantification: Box–plots of the quantification of (**A**) helper, (**C**) cytotoxic, (**E**) double-negative, and (**G**) double–positive T lymphocytes in the studied groups. Red lines indicate the median cell count in no HIV/primary EBV individuals. #: high score in relation to no HIV/primary EBV individuals. *: high count among those infected. ###: *p* < 0.001; # *: *p* < 0.05. Cartesian graph of the regression between EBV viral load and the quantification of (**B**) helper, (**D**) cytotoxic, (**F**) double-negative, and (**H**) double–positive T lymphocytes in the studied groups.

The median number of helper T lymphocytes was higher only in the no HIV/primary EBV group when compared to others [*p* < 0.0001; (HIV: med: 317.5 IQ (25–75%): 165.25–499.25); (HIV/EBV-1: med: 374 IQ (25–75%): 120.429); (HIV/EBV-2: med: 456 IQ (25–75%): 215–534); (HIV/EBV-1/2: med: 134 IQ (25–75%): 115–217); (no HIV/primary EBV: med: 1009 IQ (25–75%): 795–1218)] (Figure 3A). The regression of the EBV viral load in relation to the CD4^+^ T count was significant only in the HIV/EBV-1/2 group, in which we observed a logarithmic decrease in the multi-infection viral load as the lymphocyte count increased (*p*: 0.04; R2: 0.80). There was a trend of exponential increase in EBV viral load as a function of helper T lymphocytes in the HIV/EBV-2 group (*p*: 0.09; R2: 0.66). We did not observe a correlation between the EBV viral load and the CD4^+^ T count in the HIV/EBV-1 group. The closest mathematical regression obtained was a geometric function (*p*: 0.62; R2: 0.037) (Figure 3B).

The median cytotoxic T lymphocyte count was lower only in the no HIV/primary EBV group when compared to others [*p* < 0.0001; (HIV: med: 1050 IQ (25–75%): 737.25–1442.25); (HIV/EBV-1: med: 1015 IQ (25–75%): 951–1959); (HIV/EBV-2: med: 1127 IQ (25–75%): 854–2819); (HIV/EBV-1/2: med: 987 IQ (25–75%): 638–1034); (no HIV/primary EBV: med: 561; IQ (25–75%): 450–745)] (Figure 3C). We did not find a correlation between the EBV viral load and the cytotoxic T lymphocyte count in the coinfected groups; there were only trends of a linear increase in the viral load as a function of cell count in the HIV/EBV-1 group (*p*: 0.25; R2: 0.19), an exponential decrease in the HIV/EBV-2 group (*p*: 0.24; R2: 0.42), and a linear decrease in the HIV/EBV-1/2 group (*p*: 0.56; R2: 0.12) (Figure 3D).

The number of DN T lymphocytes was higher in the HIV/EBV-2 group when compared to the others [(*p*: 0.046; (HIV: med: 65 IQ (25–75%): 39–101.25); (HIV/EBV-1: med: 60 IQ (25–75%): 47–95); (HIV/EBV-2: med: 116 IQ (25–75%): 91–141); (HIV/EBV-1/2: med: 31 IQ (25–75%): 18–54); (no HIV/primary EBV: med: 76; IQ (25–75%): 56–108)] (Figure 3E). We did not find a correlation between the EBV viral load and the DN count; there were only trends of a linear increase in the viral load as a function of cell count in the HIV/EBV-1 group (*p*: 0.16; R2: 0.26), a logarithmic increase in the HIV/EBV-2 group (*p*: 0.92; R2: 0.042), and a linear decrease in the HIV/EBV-1/2 group (*p*: 0.60; R2: 0.10) (Figure 3F).

The number of DP T lymphocytes was higher in the HIV/EBV-1 group when compared to the others [(*p*: 0.056; (HIV: med: 4 IQ (25–75%): 2–8); (HIV/EBV-1: med: 7; IQ (25–75%): 4–9); (HIV/EBV-2: med: 3 IQ (25–75%): 2–6); (HIV/EBV-1/2: med: 3 IQ (25–75%): 1–3); (no HIV/primary EBV: med: 10; IQ (25–75%): 5–16)] (Figure 3G). A significant regression was observed only in the HIV/EBV-1/2 group, i.e., a decreasing inversely proportional relationship between the viral load and lymphocyte count (*p*: 0.001; R2: 0.96); there were only nonsignificant trends of a linear increase in viral load as a function of the lymphocyte count in the HIV/EBV-2 group (*p*: 0.10; R2: 0.64) and a geometric trend for the variables in the HIV/EBV-1 group (*p*: 0.00; R2: 0.96) (Figure 3H).

**Figure 4:**
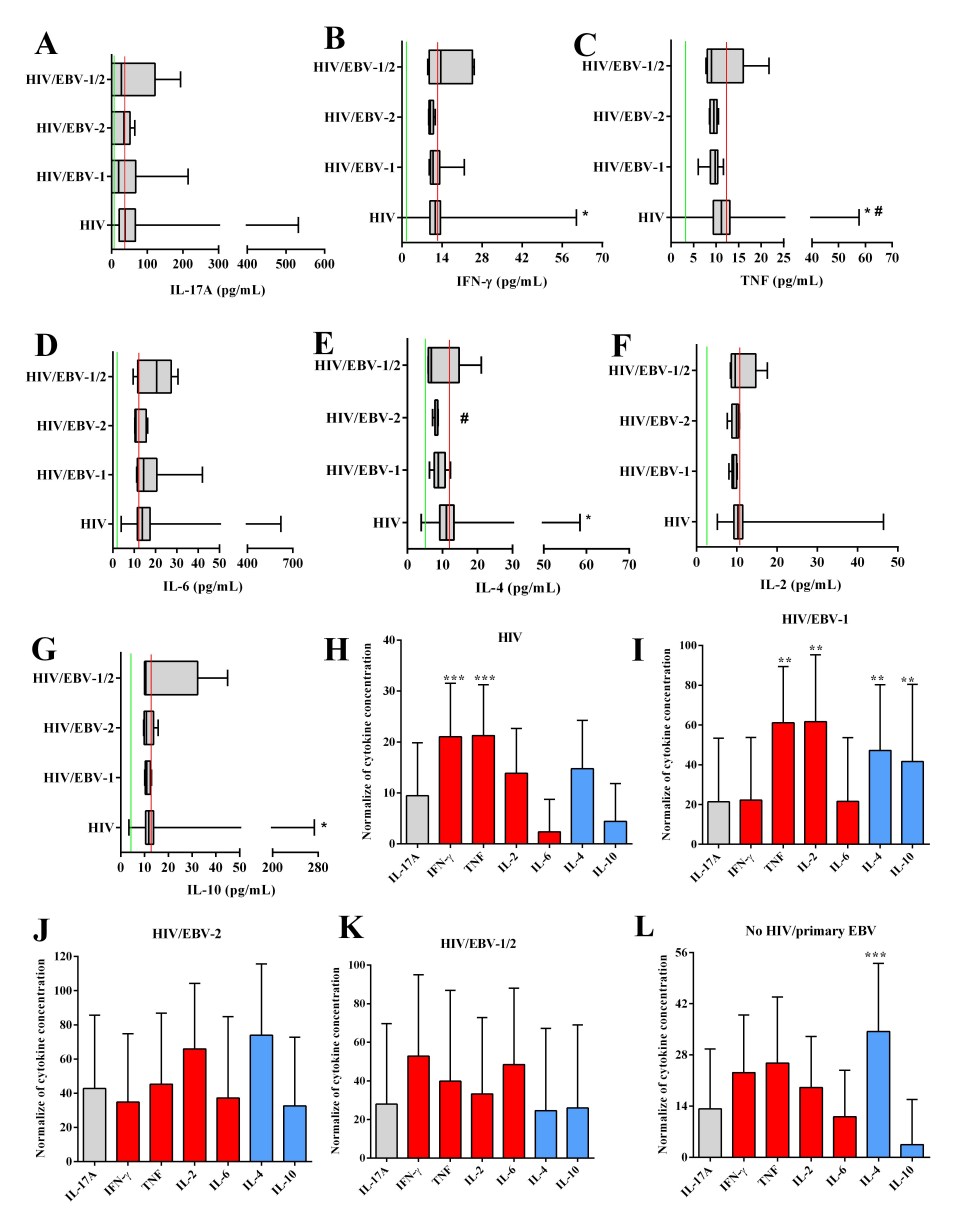
Cytokine levels: Box–plots of the cytokines IL-17A (**A**), IFN-γ (**B**), IL-4 (**C**), TNF (**D**), IL-6 (**E**), IL-2 (**F**), and IL-10 (**G**). Red lines indicate the median cytokine levels in the no HIV/primary EBV group. Green lines indicate the limit of detection for each cytokine defined by the assay manufacturer (IL-17A: 18.9 pg/mL; IFN-γ: 3.7 pg/mL; IL-4: 4.9 pg/mL; TNF: 3.8 pg/mL; IL-6: 2.4 pg/mL; IL-2: 2.6 pg/mL; IL-10: 4.5 pg/mL). #: different levels compared to the no HIV/primary EBV group. *: different levels among the infected groups. Graph of the comparison of immunological profiles based on the normalized concentration of cytokines evaluated in the HIV (**H**), HIV/EBV-1 (**I**), HIV/EBV-2 (**J**), HIV/EBV-1/2 (**K**), and no HIV/primary EBV (**L**) groups. Gray: TH17 cytokines; red: TH1 cytokines; blue: TH2 cytokines. *: *p* < 0.05; **: 0.005 > *p* > 0.001; ***: *p* < 0.001.

In the analysis of cytokine concentrations, the median concentrations IFN-γ levels were lower in the HIV/EBV-2 group when compared to the others [(*p*: 0.051; (HIV: med: 11.66 IQ (25–75%): 9.80–13.48); (HIV/EBV-1: med: 10.92 IQ (25–75%): 10.14–11.94); (HIV/EBV-2: med: 3 IQ (25–75%): 2–6); (HIV/EBV-1/2: med: 13.57 IQ (25–75%): 10.24–24.02); (no HIV/primary EBV: med: 12.61; IQ (25–75%): 10.87–14.19)] (Figure 4B).

The no HIV/primary EBV group had higher TNF levels than the other groups [(*p*: 0.002; (HIV: med: 11.16 IQ (25–75%): 9.37–13.01); (HIV/EBV-1: med: 9.77 IQ (25–75%): 9.25–10.24); (HIV/EBV-2: med: 9.52 IQ (25–75%): 8.72–9.91); (HIV/EBV-1/2: med: 8.98 IQ (25–75%): 8.33–10.30); (no HIV/primary EBV: med: 12.065; IQ (25–75%): 10.16–14.02)] (Figure 4C).

HIV/EBV-2 coinfected individuals had the lowest IL-4 values among all groups [(*p*: 0.0001; (HIV: med: 11.14 IQ (25–75%): 9.24–13.27); (HIV/EBV-1: med: 8.82 IQ (25–75%): 8.52–9.79); (HIV/EBV-2: med: 8.61; IQ (25–75%): 8.52–8.62); (HIV/EBV-1/2: med: 6.79 IQ (25–75%): 6.07–8.42); (no HIV/primary EBV: med: 12.78; IQ (25–75%): 10.07–14.28)] (Figure 4E).

The evaluation of normalized cytokine data indicated the prevalence of the TH1 profile in the HIV mono-infected group, with higher levels of IFN-γ cytokines (*p* < 0.0001; med: 19.12; IQ (25–75%): 16.07–22.10) and TNF (*p* < 0.0001; med: 19.36; IQ (25–75%): 16.26–22.57) (Figure 4H), and codominance of the TH1 and TH2 profiles in the HI/EBV-1 group, with higher levels of TNF (*p*: 0.0035; med: 66.67; IQ (25–75%): 57.38–75.04), IL-2 (*p*: 0.0035; med: 57.00; IQ (25–75%): 41.06–92.75), IL-4 (*p*: 0.0035; med: 41.83; IQ (25–75%): 36.83–58.00) and IL-10 (*p*: 0.0035; med: 29.33; IQ (25–75%): 9.89–77.74) (Figure 4I). We did not observe a prevalence of immunological profiles in the HIV/EBV-2 and HIV/EBV-1/2 coinfected groups, and all cytokines had similar concentrations (Figure 4J,K). In the no HIV/primary EBV group, compared with the other cytokines, the IL-4 levels were highest (*p* < 0.0001; med: 35.86; IQ (25–75%): 21.45–45.00) (Figure 4L).

**Figure 5:**
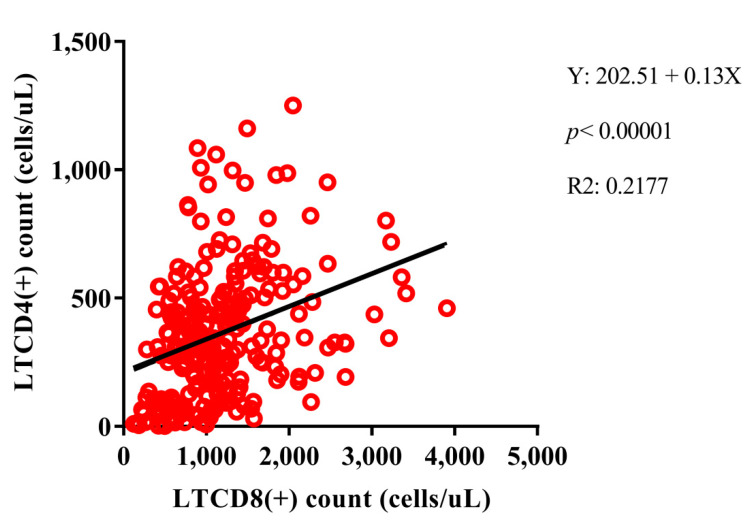
Positive linear regression model between helper T lymphocyte (LTCD4+) and cytotoxic T lymphocyte (LTCD8+) counts among PLHIV no primary EBV infection.

**Figure 6:**
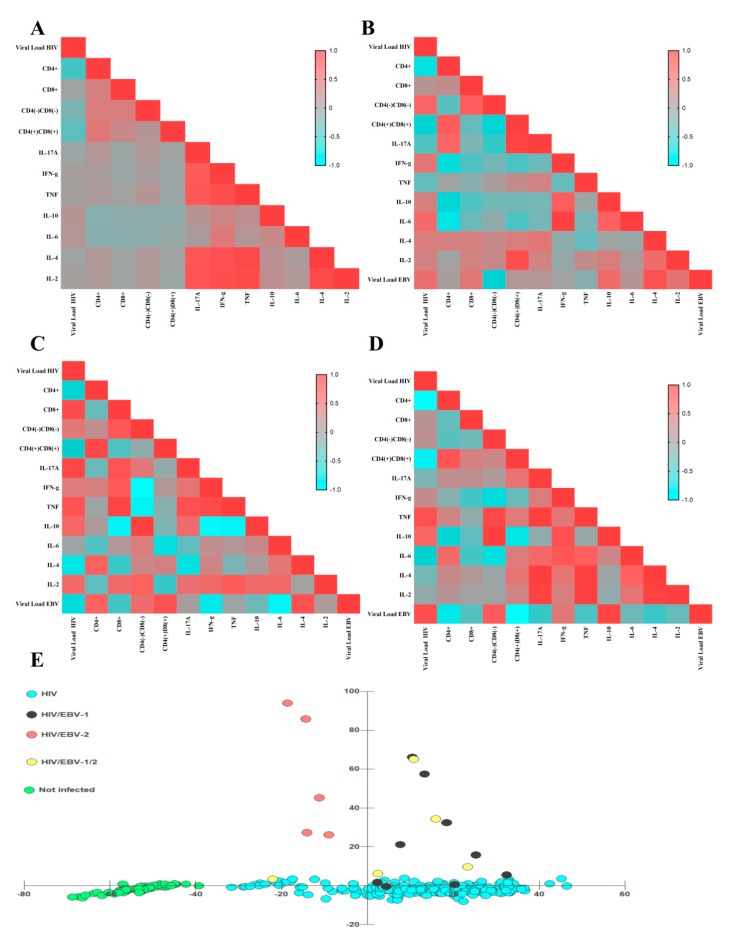
Matrix of correlations and clusters. (**A**–**D**) Heatmap graphs illustrating the Pearson coefficient of each analyzed correlation between immunological and virological factors for different groups: (**A**) PLHIV no primary EBV infection; (**B**) HIV/EBV-1; (**C**) HIV/EBV-2; (**D**) HIV/EBV-1/2. The color spectrum varies from blue (negative correlations) to red (positive correlations), as per the legend in the figures. (**E**) Discriminant dispersion diagram of the studied groups based on their immunological and viral load profiles. The HIV/EBV-2 group did not overlap with the coinfected cluster.

In the group PLHIV no primary EBV infection, HIV-1 viral load was positively correlated with IL-10 (*p*: 0.04, r: 0.12) and IL-6 (*p*: 0.05, r: 0.11) dosages, and negatively correlated with helper T lymphocytes (*p* < 0.0001, r: −0.38), DN T lymphocytes (*p*: 0.003, r: −0.19) and DP T lymphocytes (*p* < 0.0001, r: −0.30). Interestingly, helper T lymphocyte counts were positively correlated with cytotoxic T lymphocytes, with the linear graph being the best regression model among the data (*p* < 0.0001, R2: 0.2177) (Figure 5), DN T lymphocytes (*p* < 0.0001, r: 0.39), and DP T lymphocytes (*p* < 0.0001, r: 0.43), such as IL-17A dosages (*p*: 0.05, r: 0.12); and negatively correlated with IL-10 (*p*: 0.03, r: −0.14) and IL-6 (*p*: 0.04, r: −0.13) dosages. The cytotoxic T lymphocyte count was correlated only with the T DN (*p* < 0.0001, r: 0.37) and T DP (*p* < 0.0001, r: 0.26) lines. The strongest system correlations were observed between cytokines, with p values ranging from 0.04 to <0.0001 and r values ranging from 0.10 to 0.90; only IL-6 was only correlated with IFN-γ (*p* < 0.0001, r: 0.40) and IL-10 (*p*: 0.003, r: 0.24) (Figure 6A).

In the HIV/EBV-1 group, HIV viral load was positively correlated with IL-6 dosage (*p*: 0.04, r: 0.58) and IFN-γ (*p*: 0.0, r: 0.45), as was the EBV viral load (*p*: 0.03, r: 0.52); and negatively correlated to helper T lymphocyte counts (*p*: 0.04, r: −0.69) and DP T lymphocytes (*p*: 0.04, r: −0.52). EBV viral load was positively correlated with IL-4 dosage (*p*: 0.001, r: 0.82) and IL-10 (*p*: 0.03, r: 0.56). Helper T lymphocyte count was positively correlated with DP T lymphocytes (*p*: 0.03, r: 0.72), as was IL-17A dosage (*p*: 0.05, r: 0.61); and negatively correlated with IFN-γ dosage (*p*: 0.05, r: −0.63), and IL-6 (*p*: 0.03, r: −0.71). The cytotoxic T lymphocyte count was correlated only with the T DN lineage (*p*: 0.04, r: 0.67). PD T lymphocyte count was positively correlated with IL-17A dosage (*p* < 0.0001, r: 0.95) and IL-2 (*p*: 0.002, r: 0.84). Among cytokines, IFN-γ dosage was correlated to IL-10 (*p*: 0.04, r: 0.69) and IL-6 (*p* < 0.0001, r: 0.97); IL-6 dosage was also correlated with IL-10 (*p*: 0.05, r: 0.66) (Figure 6B).

In the HIV/EBV-2 group, HIV viral load was positively correlated with cytotoxic T lymphocyte count (*p*: 0.05, r: 0.87) and IL-17A dosage (*p*: 0.03, r: 0.92); and negatively correlated with T helper lymphocyte count (*p*: 0.03, r: −0.57) and EBV viral load (*p*: 0.02, r: −0.62). EBV viral load was positively correlated with T helper lymphocyte counts (*p*: 0.05, r: 0.65); and negatively correlated with IL-6 (*p* < 0.0001, r: −0.91) and IFN-γ (*p*: 0.005, r: −0.751) dosages. Helper T lymphocyte count was positively correlated with DN T lymphocytes (*p*: 0.04, r: 0.85) and negatively correlated with cytotoxic T lymphocytes (*p*: 0.02, r: −0.28). Cytotoxic T lymphocyte count was positively correlated with TNF assay (*p*: 0.03, r: 0.92) and negatively correlated with IL-10 (*p*: 0.04, r: −0.89). DN T lymphocyte count was positively correlated with IL-10 dosage (*p*: 0.01, r: 0.95) and negatively correlated with IFN-γ dosage (*p*: 0.003, r: −0.98) and TNF (*p*: 0.05, r: −0.86). Among cytokines, IL17-A was correlated with IFN-γ dosage (*p*: 0.005, r: 0.81); IFN-γ was correlated to TNF (*p*: 0.01, r: 0.87) and with IL-10 (*p*: 0.002, r: −0.93); TNF was also correlated with IL-10 (*p*: 0.03, r: −0.91) (Figure 6C).

In the HIV/EBV-1/2 group, HIV viral load was positively correlated with EBV viral load (*p*: 0.05, r: 0.85) and TNF dosage (*p*: 0.009, r: 0.79), and negatively correlated to helper T lymphocyte counts (*p*: 0.003, r: −0.98) and DP T lymphocytes (*p*: 0.05, r: −0.85). EBV viral load was positively correlated with IL-10 dosage (*p*: 0.05, r: 0.92) and negatively correlated with helper lymphocyte count (*p*: 0.02, r: −0.78), DN T lymphocytes (*p*: 0.05, r: −0.32) and DP T lymphocytes (*p*: 0.002, r: −0.98). Helper T lymphocyte count was negatively correlated with EBV viral load (*p*: 0.05, r: −0.77). Cytotoxic T lymphocyte count was positively correlated with DN T lymphocyte count (*p*: 0.02, r: 0.94). Among the cytokines, IL-17A dosage was correlated to TNF (*p*: 0.009, r: 0.96), IL-4 (*p*: 0.0007, r: 0.99), and IL-2 (*p*: 0.01, r: 0.95); TNF was correlated to IL-6 (*p*: 0.005, r: 0.98) and IL-4 (*p*: 0.006, r: 0.97); IL-4 was correlated to IL-2 (*p*: 0.02, r: 0.94) (Figure 6D).

In the dispersion diagram, the grouping of no HIV/primary EBV individuals was the most distinct among the others. Among the infected patients, we observed an overlap of the profiles for the HIV/EBV-1 and HIV/EBV-1/2 groups, which tended to be close to those mono-infected with HIV; the HIV/EBV-2 coinfected patients were grouped independently of the others and without points of intersection (Figure 6E).

### 3.4. Late Attendance and Symptomatology Affect the Profile of Immunological and Virological Markers

We calculated the time of late attendance of the HIV-1-infected patients and approximately 40% of multi-infected patients were more than 12 months after the first diagnosis and had not started antiretroviral treatment, a statistically significant proportion when compared to the delay time of the other groups (G test: 27.47; *p*: 0.001) (Table 1).

**Figure 7:**
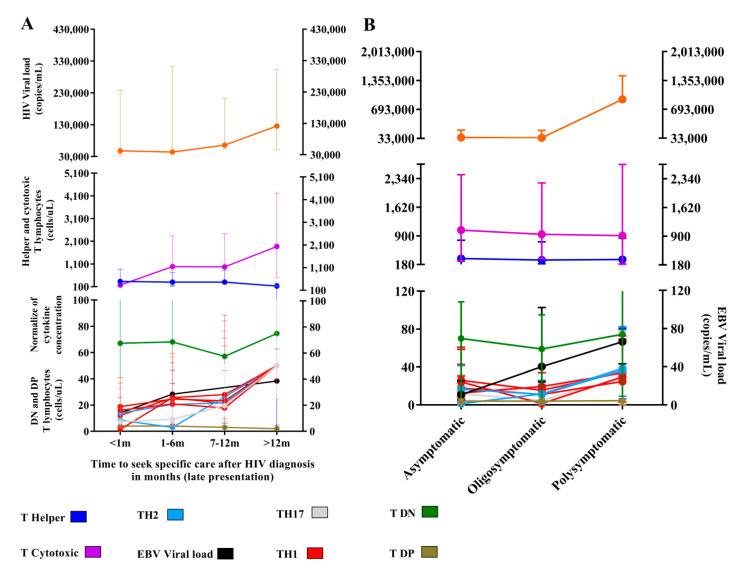
Time of seeking specific care and symptomatology: (**A**) Graph of the evaluation of immunological markers and viral load after different delays in attendance. In total, 129 patients with immediate attendance (<1 m), 105 patients with attendance between 1 to 6 months (1–6 m), 32 patients with attendance between 7 to 12 months (7–12 m) and 4 patients with late attendance (>12 m), were evaluated. (**B**) Graph of the evaluation of immunological markers and viral loads based on symptomatologic category. Totals of 138 asymptomatic, 74 oligosymptomatic, and 56 polysymptomatic patients were evaluated.

We evaluated the profile of immunological markers based on the delay in time of attendance of patients (Figure 7A). We observed a subtle increase in the median HIV viral load in patients with more than 12 months of delay after the first diagnosis (*p*: 0.9636; med: 125704.5; IQ (25–75%): 74149.25–177259.75). Regarding EBV, there was a linear increase in viral load based on the delay of adherence, with a peak in patients more than 12 months after the first diagnosis (med: 38.3; IQ (25–75%): 24.17–52.50), but we did not observe significant differences between the medians of sample collections (*p*: 0.7713). The median T helper lymphocyte count remained below 400 cells/µL at all adherence times, and only the cytotoxic T lymphocyte count changed; it was higher in patients more than 12 months after the first diagnosis (*p*: 0.00034; med: 1870.5; IQ (25–75%): 1383.75–2357.25). The number of DN T lymphocytes was lower in patients with a delay of 7 to 12 months (med: 57.00; IQ (25–75%): 27.00–79.00), but the difference was not statistically significant (*p*: 0.3913). The DP T lymphocyte count was similar between the different times of attendance (*p*: 0.7208).

There was a prevalence of the TH1 profile in patients with recent presentation, particularly the IFN-γ level (*p*: 0.0004; med: 18.8; IQ (25–75%): 16.30–22.17); the codominance of all profiles was observed in patients 7 to 12 months after the first diagnosis, and greater evidence was obtained for patients more than 12 months after the first diagnosis.

The symptoms were similar among the groups; symptoms such as fever and sore throat were the most frequent (Table 1). We grouped the patients as asymptomatic, oligosymptomatic (two to three symptoms), and polysymptomatic (four or more symptoms). Asymptomatic patients were more frequent, except for multi-infected patients, for whom the proportion of oligosymptomatic patients was more evident; however, the difference was not significant (G test: 06.09; *p*: 0.41) (Table 1).

We also analyzed the fluctuations in the biomarkers of HIV-1 and EBV infections based on symptomatologic groups (Figure 7B). The median HIV viral load was higher in polysymptomatic patients (*p*: 0.04; med: 45,998.5; IQ (25–75%): 12,936.25–173,970.5). The counts of CD4^+^ T and CD8^+^ T cells remained similar among all groups, and the median CD4^+^ T count remained below 310 cells/µL in all groups. The EBV viral load was lower in asymptomatic patients (*p*: 0.05; med: 10.67; IQ (25–75%): 5–14.67) and gradually increased in oligo- and polysymptomatic patients. We did not observe significant differences in the SD or DN T lymphocyte count (*p*: 0.5787, *p*: 0.2335, respectively). We observed a predominance of the TH1 profile in asymptomatic (*p*: 0.0004) and oligosymptomatic (*p*: 0.0001) patients, although a decrease in cytokine levels occurred in oligosymptomatic patients, with the exception of IL-10, which was increased in asymptomatic patients (*p* < 0.0001; med: 11.55; IQ (25–75%): 9.71–14.18); in the polysymptomatic group, codominance of all profiles prevailed. We emphasized the increased levels of IL4 (*p* < 0.0001; med: 36.28; IQ (25–75%): 27.06–43.12), IL-10 (*p* < 0.0001; med: 38.4; IQ (25–75%): 32.26–44.12) and IL-17A (*p*: 0.0019; med: 29.29; IQ (25–75%): 19.73–48.67).

## 4. Discussion

The low overall prevalence of primary EBV infection was similar to that observed in other developing countries [17,18]. However, we expected that the rate of coinfected patients among the immunocompromised patients would be higher [19]; these divergences may be related to the methodology used. In the present study, the anti-VCA IgM (+) IgG (−) serological panel was used for sample screening; this profile is indicative of primary EBV infection, has high sensitivity, and is applied in the discrimination of false-positive cases [20].

In the surveillance of EBV infection, there is controversy as to the type of blood extract that should be used as a standard in determining viral load. The quantification of different fractions may provide different information that may or may not correspond to the clinical profile of the patient [21]. In the present study, we quantified the viral load of EBV in the buffy coat and blood plasma, and discrepant levels of viral genetic material were observed in the buffy coat samples; however, all associations were established using the viral load in blood plasma.

There is no methodological consensus regarding the best extract in cases of primary infection or infectious mononucleosis. It has been suggested that most of the EBV load is located in the cell fraction and that low detection in plasma is related to the release of viral DNA from apoptotic cells or damage to infected B lymphocytes during the separation of blood compartments [22]. We confirmed that the viral load was high in the buffy coat. However, it is argued that the detection of EBV in plasma is a biomarker of active and replicative infection due to the rare circulation of free viral particles in the plasma; therefore, any change in this parameter is indicative of infection [23,24].

Therefore, we suggest that plasma may be a more promising sample for assessing primary EBV infection, consistent with results from the aforementioned studies. Cell extracts are more convenient for determining clinical profiles when the virus is latent, as in cases of lymphomas associated with infection [25,26].

Regarding the virological interaction, we observed that the EBV-2 viral load was associated with low plasma levels of HIV viral load, with trends toward the maintenance of the helper T lymphocyte count and the control of cytotoxic T lymphocytes. The coinfected group exhibited low levels of plasma cytokines, especially IFN-γ and IL-4, without dominance of effector immunological profiles.

The EBV-2 viral load was inversely associated with IL-6 and IFN-γ levels. Studies have shown that the viral protein LMP2a regulates the production of IL-6 [27] and decreases the capacity of the cellular response to stimulation by IFN-γ [28]. Other gene products interfere with the transcriptional pathway of factors linked to IFN production and the induction of an antiviral state [29].

IL-4, although at significantly low levels, was related to the EBV-2 viral load, a finding that may have implications both in maintaining an anti-inflammatory microenvironment [30] and in favoring viral persistence [31], and affected cell susceptibility by inducing the expression of surface markers [32]. We suggest that in coinfection, EBV-2 modulates the immune response, leading to a homeostatic threshold that contributes to the regulation of inflammation, which consequently contrasts with the overall inflammatory state of the progression of HIV infection [33], as observed in the present study. It is likely that the positive correlation between T helper and cytotoxic T lymphocyte counts observed herein reflects a marked cellular response that results in partial recovery of T helper lymphocytes [34].

Another noteworthy point is the indirect association of EBV-2 with the number of DN T lymphocytes. In our findings, these cells were strongly associated with IL-10 levels, which may contribute to the modulation of inadequate immune activation and limit the tissue damage characteristic of HIV infection [35]. This could also explain the negative correlation between DN T lymphocytes and cytotoxic T lymphocytes through TNF, which is a factor that promotes clonal expansion and differentiation and the survival of cytotoxic lymphocytes [36]. In simian retrovirus (SIV) infection models, it was found that in the depletion of helper T lymphocytes, DN T lymphocytes emerge as a potential subset of cells capable of performing similar functions [37]. In the present study, both strains were favored by the immunomodulation suggested for EBV-2.

We did not rule out a specific association between EBV-2 and T lymphocytes because this particular genotype is able to infect these cells and use them as a persistent or additional latent reservoir [38], which is also observed in the context of coinfection with HIV [39]. The infection of T cells makes them both a replicative niche supporting primary infection or reactivation and microenvironments conducive to viral latency, leading to the proliferation of infected T lymphocytes with the consequent control of the immune response [40]. It has not been established which T cell lines are most affected by EBV-2 infection; however, we suggest that DN T lymphocytes may be the target cells in this process because they were the lineage with a significantly higher absolute number in the HIV/EBV-2 coinfected group, a finding that has been observed in other clinical profiles associated with EBV [41]. Thus, the supply of T lymphocytes with auxiliary functions affected by EBV-2 favors the immune balance of the anti-HIV response.

From a cellular point of view, studies indicate that in transformed B lymphocyte lines, the regulation of the EBNA2 gene, and probably LMP1, of EBV-2 [42] is associated with low expression of CCR5 [43] and CXCR4 [44,45] and HIV entry receptors in the host cell [46]. Although these results pertain to B cells, the changes induced by herpesvirus were associated with the selection of HIV strains by receptor tropisms [47], and we cannot rule out that these changes may occur at the level of T lymphocytes because EBV-2 can also infect these cells.

Notably, all the likely modifications induced by EBV-2 favor the pathogenesis of the virus. Thus, longitudinal studies of HIV/EBV-2 coinfection are needed to evaluate, over the long term, the repercussions of the viral scenario observed in the present study and whether it can contribute to the typically observed herpesvirus lymphomagenesis [47].

In contrast to what was found in the HIV/EBV-2 group, our results suggest a positive correlation between the EBV-1 infection markers and the HIV-1 infection biomarkers. Certainly, the correlation of the virological markers of both agents suggests that the persistence of coinfection favors the expansion of infected cells and the onset of related malignant diseases [48]. Recent results show that under conditions of impaired immune response, EBV-1-infected B lymphocyte lines are susceptible to HIV-1, which could potentially reside in the latent form in memory B cells and thus constitute a potential additional reservoir for infection [49].

In our findings, the viral interaction in HIV/EBV-1 coinfection may have occurred through a proinflammatory pathway due to the correlation of HIV viral load with IL-6 and IFN-γ, potentially resulting in the plausible depletion of helper T lymphocytes, which is a well-known profile for the attempted control and suppression of the most evident infection in the early stages of the disease [50]. Alternatively, EBV viral load was directly proportional to IL-4 and inversely proportional to IL-10, potentially counteracting the exacerbated and harmful inflammation initiated by the type 1 response [51]. This distorted immune balance contributes to the maintenance of HIV infection, as observed in other clinical profiles associated with infection [52], whose predominance of a proinflammatory response similar to that observed has also been associated with the reactivation of EBV in more severe states of retroviral immunodeficiency [53]. In fact, we observed that HIV alone could illicit a multi-interactive immune control system that culminated in the predominance of an inflammatory profile.

In this study, the DP T lymphocyte count was high only in the HIV/EBV-1 group and had no direct association with the agents themselves, but was a consequence of the change in the number of T helper lymphocytes resulting from the complex interactive immunopathogenic system. DP cell numbers were proportional to the levels of IL-17A and IL-2 in coinfection, a finding that may be indicative of an attempt to maintain the immune response under the imminent instability of T helper lymphocytes considering that the DP line represents a frequent and highly reactive cell population, some of which are IL-2+ subpopulations, in HIV infection [54,55]. This reinforces our hypothesis regarding the activation of a cellular response. In turn, high levels of IL-17A may help control the aggressive immune activation observed in HIV+ patients [56].

The function of DP T lymphocytes in EBV infection is not known. A baseline study detected the presence of a subpopulation with low CD4 surface expression at the onset of acute infection; however, the functions of this subpopulation have not been elucidated [57]. More recent studies have characterized this specific line as constitutively expressing high concentrations of immunological activation markers [58] in different stages of chronic viral infection. It is likely that these cells support the antiviral response through the secretion of proinflammatory cytokines and the conversion to immunological memory profiles [59].

In HIV/EBV-1/2 multi-infection, we observed an immunological profile similar to EBV-1 coinfection. In this group, the viral interaction was stronger and inversely proportional to the number of helper T lymphocytes; as an example, there was a significant increase in the levels of cytokines and a balance in the response profiles. Similarly, HIV viral load was correlated with proinflammatory cytokines, while EBV viral load was correlated with IL-10 levels.

EBV-1 prevails in cases of coinfection by both genotypes, and epidemiological changes may occur through an intense specific-type immune response [60]. Whether the distinct pathological and functionally more aggressive profile of EBV-1 may be associated with its prevalence in several clinical cases of infection has been discussed [61,62]. Recent results have shown similarities in the immunological response against EBV vaccine antigens in children infected by genotype 1 or both genotypes [63].

The heterogeneity between the EBV genotypes is not restricted to the scores in the viral genome but affects the recognition of different types in the development of the immune response [64]. New studies focused on response mechanisms to the intraspecific divergences of EBV may elucidate the pathological profiles of herpesviruses. In this study, we utilized additional data on viral interactions in the context of HIV coinfection.

We observed that the later the attendance of HIV-1-infected patients to adequate medical care, the greater the increase in cytotoxic T lymphocyte count and cytokine levels, which was associated with a slight decline in helper T lymphocytes, whose levels remained below 400 cells/µL in all groups. From the analysis of the delay in patient adherence, it is likely that the patients were in the early stages of HIV infection, which is characterized by a progressive immune response and by the inversion of the CD4^+^/CD8^+^ ratio [65], generating a vigorous cytotoxic cell response correlated with high viral loads [66], which is a viremic trait that persisted in our study.

However, in patients with late presentation, a numerical increase in HIV viral load may reflect the initial cytotoxic response, which, although following a pattern of immunodominance against some viral epitopes, is successfully predicted to some extent [67] because with ongoing escape mutations, HIV evades recognition by the cellular response [68]. Similarly, the EBV viral load tends to slightly increase, even proportional to the increase in cytotoxic T lymphocytes; this suggests that the paradoxical success of viral replication against the sustained immune response may be the result of the actions of multiple products of lytic viral genes that inhibit antigen presentation pathways [69].

The prevalent TH1 profile in patients with immediate adherence is again consistent with early and continuous proinflammatory immune activation at the beginning of coinfection [70,71]. However, long-term delays manifest as immunoregulatory profiles that interfere with the activation of the cellular response at the expense of establishing viral persistence [69,72]. The curious upregulation of all cytokine profiles in patients with longer delays in adherence may be related to the immune balance against active coinfection, which originates from both the host response and viral escape mechanisms [73,74]. Associated with these aspects, a high percentage of multi-infected patients sought specific care only 12 months after the first HIV-1 diagnosis. This highlights our concern because this infectious profile was associated with favoring HIV pathology, and delay in care was detrimental to the maintenance of the antiviral response.

Asymptomatic individuals prevailed in all groups. The symptoms, when present, were similar to both the initial phase of HIV infection and the primary symptomatic EBV infection [34,75]. Particularly with EBV, these symptoms were associated with age groups above 18 years and with the host’s compromised immune status [76].

Although we did not associate symptomatology with the groups studied, based on our analyses, we assume that symptomatology was more associated with EBV due to the proportionality of the viral load to the emerging symptoms [77]. These manifestations usually regress in a short time. However, in rare cases, they present heterogeneous conditions that may persist or recur repeatedly due to viral load [78]. In the face of HIV coinfection, our data suggest caution, because these conditions may be intensified by pro-pathological viral interactions [79], which would explain the peak HIV viral load associated with polysymptomatic patients.

The prevalence of the cytotoxic response with a reduced number of circulating helper T lymphocytes was expected. It is argued that EBV favors a TH1 reaction by modulating specific cytokines; consequently, CD8^+^ T cells are more numerous in the early stages of infection [80], as observed in asymptomatic and oligosymptomatic patients. We suggest that the increase in the levels of anti-inflammatory cytokines in polysymptomatic individuals may reflect the immune-mediated suppression that is characteristic of an ineffective antiviral response [81], which is consistent with the cascade of interactions proposed specifically for coinfection with EBV-1 and in multi-infection. We argue that this complex viral interaction also impacts the maintenance of the HIV viral load in polysymptomatic patients because the significant increase in this marker is a predictor of disease progression and clinical worsening [82], which plays a critical role in the escape of immune recognition via multifaceted mechanisms [83], potentially contributing to the pathological scenario.

## 5. Conclusions

We conclude that different immunological profiles are associated with the EBV genotype in primary EBV infection in untreated PLHIV. The results suggest that EBV-2 was associated with low levels of HIV-1 viral load, whereas EBV-1 viral load was positively correlated with HIV-1 viral load. These aspects become more worrying with a delayed attendance to specific care and the consequent delay in adherence to antiretroviral treatment, which leads to clinical changes and effects on the maintenance of the immune response. Finally, although the size of our sample of infected patients was small, it is probably a reflection of the real-world prevalence of primary EBV infection in adult PLHIV in the Brazilian Amazon region.

## Figures and Tables

**Table 1:** Sample characterization of the studied groups according to sex, age, delay in seeking specific care, and reported symptoms.

Characteristics	PLHIV no Primary EBV	HIV/Primary EBV 19 (7.09)			No HIV/Primary EBV	Statistic
		HIV/EBV-1	HIV/EBV-2	HIV/EBV-1/2		
**Sample number**	249	9 (47.37)	5 (26.32)	5 (26.32)	65	
Sex						
Feminine	49 (19.7)	5 (56.0)	1 (20.00)	1 (20.0)	17 (26.2)	06.22 (0.23) ^#^
Masculine	200 (80.3)	4 (44.0)	4 (80.00)	4 (80.0)	48 (73.8)	
**Age (years)**						
18–38	186 (74.70)	8 (88.89)	3 (60.00)	5 (100.00)	43 (66.15)	07.31 (0.82) *
39–59	59 (23.69)	1 (11.11)	2 (40.00)	0	20 (30.77)	
60–80	4 (01.61)	0	0	0	2 (03.08)	
**Delay in seeking assistance (months)**						
<1 m	122 (49.00)	2 (22.0)	4 (80.0)	1 (20.0)	-	27.47 (0.001) *
1–6 m	95 (38.00)	7 (78.0)	1 (20.0)	2 (40.0)	-	
7–12 m	32 (13.00)	0	0	0	-	
>12 m	0	0	0	2 (40.0)	-	
**Symptomatology**						
Fever	177 (71.08)	7 (77.77)	5 (100.00)	5 (100.00)	-	6.54 (0.69) *
Headache	110 (44.18)	7 (77.77)	4 (80.00)	2 (40.00)	-	
Myalgia and joint pain	90 (36.14)	5 (55.55)	1 (20.00)	0	-	
Sore throat	197 (79.12)	7 (77.77)	5 (100.00)	3 (60.0)	-	
**Groups**						
Asymptomatic	132 (53.01)	2 (22.22)	3 (60.00)	1 (20.00)	-	6.09 (0.41) *
Oligosymptomatic	67 (26.91)	3 (33.33)	1 (20.00)	3 (60.00)	-	
Polysymptomatic	50 (20.08)	4 (44.44)	1 (20.00)	1 (20.00)	-	
**Infection history**						
No	142 (57.07)	3 (42.86)	3 (60.00)	2 (40.00)	62 (95.00)	16.70 (0.67) *
Fungi	1 (00.26)	0	0	0	1 (01.67)	
Bacterial (STD)	86 (34.45)	4 (57.14)	2 (40.00)	3 (60.00)	0	
HPV	10 (4.11)	0	0	0	1 (01.67)	
Herpes	9 (3.60)	0	0	0	1 (01.67)	
Hepatitis	1 (00.51)	0	0	0	0	

^#^: Fisher’s exact test (*p*-value); *: Test G (*p* value).

## Data Availability

Data supporting the findings of this study are available from Igor Brasil Costa, but restrictions apply to the availability of these data, which were used under license for the present study and therefore are not publicly available. However, data are available from the authors upon reasonable request and with permission from Igor Brasil Costa.

## Supplementary Materials

The following are available online at https://www.mdpi.com/article/10.3390/v14020168/s1, Figure S1: Laboratory report produced by BD Multiset™ Software v3.1 (BD Biosciences, San Jose, CA, USA). Cell quantification is standardized by the manufacturer and is unique to lymphocyte populations with direct relevance to the pathogenesis of HIV infection. There was no need to change the intercept gates of dot plot graphics; Table S1: Correlation of EBV viral load quantified in plasma and whole blood with immunological markers, symptoms and time of adherence to HAART.
