# Supplementary material for: Epstein–Barr Virus (EBV) Genotypes Associated with the Immunopathological Profile of People Living with HIV-1: Immunological Aspects of Primary EBV Infection
Source: Viruses. 2022 Jan 18;14(2):168. doi: 10.3390/v14020168 (PMC8880155; doi:10.3390/v14020168)
Supplement: Supplementary file 1 [file viruses-14-00168-s001.zip › viruses-1483083-supplementary.pdf]

Supplementary Materials:

MultiSET™ Lab Report

Director:		Software:	MultiSET V3.1
Operator:	Admin	Cytometer:	FACSCalibur (#E34297502763)
Sample Name		Date Acquired:	Thu, Oct 7, 2021 11:39 AM
Sample ID:		Date Analyzed:	Thu, Oct 7, 2021
Case Number:		Ref. Range Type:	BD
Panel Name:	ROTINA 4C		

CD3/CD8/CD45/CD4 TruC

Data Set [1] Data File: 6351816.01

Reagent Lot ID: 30874 Events Acquired: 20000 Abs Cnt Bd Lot ID: 21138 Attr Def File: 3/8/45/4 MLT/TruC v2.0

File ID: 9E53585C-B461-42A1-8E5B-B1E68954EE43 Beads Per Pellet: 48300

Lymph Events	7838	
Bead Events	2266	
CD3+ %Lymph	82	
CD3+ Abs Cnt	2733	Hi
CD3+CD8+ %Lymph	51	Hi
CD3+CD8+ Abs Cnt	1709	Hi
CD3+CD4+ %Lymph	29	Lo
CD3+CD4+ Abs Cnt	979	
CD3+CD4+CD8+ %Lymph	1	
CD3+CD4+CD8+ Abs Cnt	31	
CD45+ Abs Cnt	3341	
T H/S Ratio	0.57	Lo

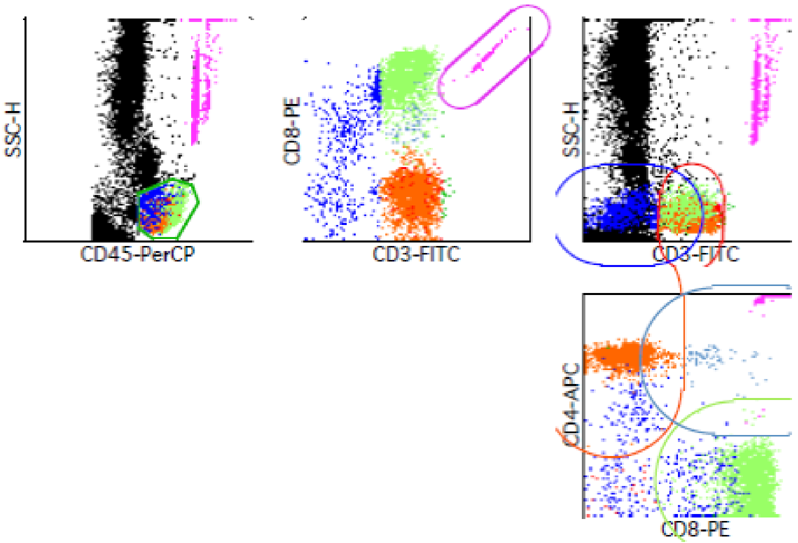

Figure S1: Laboratory report produced by BD Multiset™ Software v3.1 (BD Biosciences, San Jose, CA, USA). Cell quantification is standardized by the manufacturer and is unique to lymphocyte populations with direct relevance to the pathogenesis of HIV infection. There was no need to change the intercept gates of dot plot graphics.

Table S1. Correlation of EBV viral load quantified in plasma and whole blood with immunological markers, symptoms and time of adherence to HAART.

		EBV viral load in plasma			EBV viral load in buffy coat (log10)		
**Markers**	med (IIQ 25%-75%)	med (IIQ 25%-75%)	rs	p valor	med (IIQ 25%-75%)	rs	p valor
IL-4	8.61 (7.01-9.00)	16.67 (7.67-67.00)	0.92	0.037	3.56 (3.23-4.41)	0.26	0.274
Double positive T lymphocytes	4.00 (2.50-7.50)		0.89	0.034		-0.14	0.557
IFN-γ	10.70 (9.90-12.76)		0.88	0.049		-0.08	0.734
IL-10	10.79 (10.20-10.51)		0.79	0.066		0.22	0.374
HIV viral load (log10)	4.38 (3.76-5.54)		0.65	0.003		0.12	0.570
Symptomatology	-		0.55	0.015		0.52	0.022
T helper lymphocytes	296.00 (127.00-434.00)		-0.35	0.085		-0.10	0.680
Cytotoxic T lymphocytes	1015.00 (875.50-2033.00)		-0.24	0.323		-0.23	0.344
IL-2	9.77 (8.84-10.03)		0.18	0.440		0.20	0.407
IL-6	14.43 (11.27-19.29)		0.16	0.521		0.14	0.557
Delay time in HAART adherence	-		0.15	0.532		0.17	0.499
TNF	9.71 (8.63-10.27)		0.11	0.640		0.08	0.740
Double negative T lymphocytes	76.00 (50.50-114.00)		0.08	0.741		-0.004	0.989
IL-17A	26.11 (0-53.69)		0.02	0.945		0.06	0.809

med: Median.
IIQ: Interquartile.
rs: Spearman's Coefficient
